# Fronto-striatal dysregulation in drug addiction and pathological gambling: Consistent inconsistencies?^☆^

**DOI:** 10.1016/j.nicl.2013.02.005

**Published:** 2013-03-05

**Authors:** Eve H. Limbrick-Oldfield, Ruth J. van Holst, Luke Clark

**Affiliations:** a Department of Psychology, University of Cambridge, Cambridge, UK; b Donders Institute for Cognition, Brain and Behaviour, Radboud University, Nijmegen, The Netherlands

**Keywords:** Addiction, Pathological gambling, fMRI, Ventral striatum, Appetitive processing

## Abstract

Alterations in appetitive processing are central to the major psychological theories of addiction, with differential predictions made by the reward deficiency, incentive salience, and impulsivity hypotheses. Functional MRI has become the chief means of testing these predictions, with experiments reliably highlighting disturbances at the level of the striatum, medial prefrontal cortex, and affiliated regions. However, demonstrations of *hypo*-reactivity and *hyper*-reactivity of this circuitry in drug addicted groups are reported in approximately equal measure. Similar findings are echoed in the emergent neuroimaging literature on pathological gambling, which has recently witnessed a coming of age. The first aim of this article is to consider some of the methodological aspects of these experiments that could influence the observed direction of group-level effects, including the baseline condition, trial structure and timing, and the nature of the appetitive cues (drug-related, monetary, or primary rewards). The second aim is to highlight the conceptual traction that is offered by pathological gambling, as a model of a ‘toxicity free’ addiction and an illness where tasks of monetary reinforcement afford a more direct mapping to the abused commodity. Our conclusion is that relatively subtle decisions in task design appear capable of driving group differences in fronto-striatal circuitry in entirely opposing directions, even with tasks and task variants that look ostensibly similar. Differentiation between the psychological theories of addiction will require a greater breadth of experimental designs, with more research needed on processing of primary appetitive cues, aversive processing, and in vulnerable/at-risk groups.

## Highlights

• We outline the current psychological theories of addiction and their predictions.
• We review recent fMRI literature of substance addictions and appetitive processing.
• Reasons for opposing results (hyper- vs hypo-active reward regions) are discussed.
• Recent fMRI findings of appetitive processing in pathological gambling are reviewed.
• Pathological gambling is suggested as a prototypical addiction for imaging research.

## 1 Introduction

Current conceptualisations of drug addiction are heavily informed by the neurobiological basis of motivated behaviour, with a focus primarily on appetitive processing. Several psychological theories have been put forward to characterise the changes in appetitive processing that either predispose the addicted state, or describe the transition into drug addiction. For example, the *reward deficiency* hypothesis (Blum et al., 2012; Comings and Blum, 2000) proposes that a trait-related insensitivity to naturally-occurring reinforcement predisposes the individual to drug taking as a means of compensation. *Incentive salience* or *sensitisation* accounts (Robinson and Berridge, 1993, 2008) propose that the brain response to drugs of abuse becomes potentiated over repeated use, so that drug seeking comes to dominate goal-directed behaviour over healthy rewarded behaviours. Over the past decade, functional MRI (fMRI) studies of addicted populations have become the central means of arbitrating between these accounts, as brain reward systems can be assayed effectively with a number of popular activation probes, such as Knutson's Monetary Incentive Delay Task (MIDT) (Knutson et al., 2001). In one sense, these experiments demonstrate remarkable consistency, in that they reliably localise the dysregulation in addiction to dopamine-innervated regions in the striatum and medial sector of the prefrontal cortex (mPFC). However, the *direction* of effect is conspicuously inconsistent, with multiple, high-quality experiments indicating either *hypo*-activity or *hyper*-activity of the same reward regions (Hommer et al., 2011). The first aim of the current article is to consider some of the design features of these experiments that may determine the observed direction of effect.

The second aim is to consider the broader category of addictive disorders that will be recognised within the DSM5, which is specifically proposed to include pathological gambling (to be renamed ‘Disordered Gambling’) as the prototypical form of *behavioural addiction*. The first neuroimaging studies of pathological gambling were published in the mid 2000s (Potenza et al., 2003a, 2003b; Reuter et al., 2005), and over the past year, this field has matured with the reporting of arguably the four strongest fMRI studies to date (Balodis et al., 2012a; Miedl et al., 2012; Sescousse et al., 2010; van Holst et al., 2012b). As with the studies of drug addiction, these papers in pathological gambling have isolated striatum and mPFC regions as lying at the core of this disrupted network, but again, the direction of effects across the four studies is inconsistent. In considering these findings, we will highlight the features of pathological gambling that we believe make it a valuable experimental model for the addictions field, and the leverage that may be afforded by this illness for resolving the nature of the dysregulation in reinforcement processing in drug addiction.

## 2 Psychological theories of drug addiction

Motivational circuits were originally implicated in addiction by the observation that drugs of abuse increase dopamine transmission within these circuits (Wise, 2004). The primary focus of these theories has been on the appetitive processing that governs behavioural approach, and the inhibitory control of these approach behaviours (Bechara, 2005; Goldstein and Volkow, 2002; Jentsch and Taylor, 1999). Within this framework, addiction may be related to either an increase in approach behaviour to drug-related stimuli, or a decrease in inhibitory control. While modern conceptualisations acknowledge both processes, the alternative accounts vary in the weight they afford to each. In addition, the accounts differentially emphasise either the vulnerability factors that characterise the (premorbid) disposition to addiction, or the transitional processes from casual consumption into full-blown addiction. Critically, the theories described below make different predictions about whether addicted individuals would show an increased, normal, or decreased neural response to either addiction-related stimuli, or non-drug-related appetitive cues. Such predictions are highly amenable for testing with fMRI.

The reward deficiency hypothesis predicts that the susceptibility to addiction stems from an insensitive or ineffective dopaminergic system (Comings and Blum, 2000). In this state, natural rewards will yield only an attenuated response, such that a rewarding stimulus will not drive the dopaminergic system to the required threshold to trigger the brain's ‘reward cascade’ (Blum et al., 2012), and normal experiences would not adequately impact upon motivated behaviour. As a result, the individual would seek stronger experiences – including but by no means limited to drug taking – to drive dopamine release and activate the reward cascade. The reward deficiency hypothesis originated from genetic data showing that a variant in the dopamine D2 receptor gene (Taq1A DRD2) was more prevalent in patients with alcohol dependence (Blum et al., 1990; Noble et al., 1991) and was associated with a hypo-dopaminergic state. This genotype was subsequently linked to other addictive disorders, including pathological gambling (Comings et al., 1996, 2001). The critical hypo-dopaminergic state may also occur via environmental routes such as prolonged exposure to stress (Blum et al., 2012; Madrid et al., 2001). In humans, positron emission tomography (PET) studies have demonstrated that the dopaminergic release elicited by methylphenidate is lower in addicted individuals, compared to controls (Martinez et al., 2007; Volkow et al., 1997). However, while these results suggest a hypo-dopaminergic state in the addicted brain, causality cannot be established. The hypo-dopaminergic state may represent a pre-morbid vulnerability, or could be a consequence of chronic drug use.

A contrasting model, incentive salience, also focuses on dopaminergic signalling of approach behaviour (Robinson and Berridge, 1993, 2001, 2008), but it predicts that the addicted brain exists in a hyper-dopaminergic state. Exogenous stimulation of the dopamine system is known to cause an increase in dopaminergic activity that is resistant to habituation, unlike the response to natural rewards (Di Chiara, 1999). Through repeated administration the dopaminergic response becomes sensitised (Robinson and Becker, 1986). In addition, repeated pairing of the drug (triggering a large dopaminergic response) with associated environmental stimuli (e.g., drug paraphernalia), leads these stimuli to acquire increased salience and capture attention, over and above naturally rewarding stimuli (Robinson and Berridge, 1993). Unlike the reward deficiency hypothesis, there is no requirement for a pre-morbid abnormality in the processing of natural rewards, as the addiction develops as a consequence of exogenously-driven dopamine release. Animal models have provided much support for this model (e.g., Di Ciano, 2008; Harmer and Phillips, 1998; Taylor and Horger, 1999); for example, rats pre-exposed to cocaine showed facilitated learning when associating a novel stimulus with a conditioned reinforcer that was previously paired with cocaine (Di Ciano, 2008). However, direct evidence in humans has been less compelling. For example, PET studies indicate a *reduction* in striatal dopamine receptors in addicted individuals (Martinez et al., 2004; Volkow et al., 1990), implying a *hypo*-sensitive dopamine system. Robinson and Berridge (2008) postulate that sensitisation may only be expressed within certain psychological contexts, like the environment of habitual drug taking rather than a novel environment like a brain scanner, rendering the hypothesis difficult to test with functional neuroimaging.

The third class of model emphasises a deficiency in the top-down inhibitory control of drug-taking, with a shift in the underlying neuroanatomical focus from the striatum to the PFC (Bechara, 2005). Trait elevations in impulsivity and their neuropsychological counterpart, poor inhibitory control, may predispose initial drug experimentation as well as transitions to abuse and dependence (Verdejo-García et al., 2008). Similarly, it has been suggested that adolescence may represent a critical period of maturation, during which time increased levels of trait impulsivity leave an individual vulnerable to the development of an addiction (Chambers et al., 2003). The impulsivity hypothesis gives no particular weight to drug-related reinforcement, and thus similar changes would be expected in addiction in the processing of natural rewards. In addition, by emphasising the top-down control of responding, the impulsivity hypothesis can readily accommodate the possibility that addiction may be associated with a reduced sensitivity to *aversive* consequences, either instead of or in addition to, any alteration in appetitive processing. The mPFC has been shown to be critical to maintain successful inhibition in animal models, as lesions of this region result in increased impulsivity (Gill et al., 2010). In humans, a structural MRI study in healthy participants reported that mPFC volume in the human was correlated with measures of impulsivity (Cho et al., 2012). The impaired response inhibition and salience attribution (I-RISA) model of addiction (Goldstein and Volkow, 2002; Goldstein et al., 2009) was developed to integrate the increased salience of drug-related cues as a result of repeated drug consumption (in line with the incentive salience model), and pre-morbid deficiencies in impulsivity and top-down down control that leave an individual susceptible to addiction.

The three groups of models make differential predictions about the neural basis of addiction, and specifically about increases or decreases in reward-related activity in addicted groups relative to controls. In terms of subcortical dopaminergic activity, the reward deficiency hypothesis proposes a *reduction* in reward-related processing, which would affect drug-related and non-drug-related appetitive processing similarly. The incentive salience and impulsivity hypotheses both predict that the subcortical dopaminergic response to drug-related stimuli is *increased*; however, these two accounts differ in their predictions about the response to non-drug-related appetitive stimuli: incentive salience is effectively agnostic on such stimuli, whereas the impulsivity hypothesis predicts a generalised hypersensitivity of the subcortical reward network. In addition, the impulsivity hypothesis contains an important role for mPFC function, which should be reduced and associated with the deficient inhibitory control. The impulsivity hypothesis also best accommodates any changes in the neural response to aversive events.

While several of these predictions are intuitively opposing, one must bear in mind that addiction is a dynamic disorder with distinct temporal stages. The distinct models may preferentially explain the vulnerable state and disposition to drug initiation (reward deficiency) or the transition into compulsive drug-taking (incentive salience). Once the addiction is instantiated, there is a further cyclical pattern, from binge/intoxication to withdrawal and negative affect, to preoccupation and anticipation (Koob and Le Moal, 1997). These stages will likely affect motivational systems differently; while the ‘high’ during intoxication is characterised by increased striatal dopamine transmission (Volkow et al., 1996), and withdrawal is associated with hypo-activity of the same pathways (Martinez et al., 2004, 2005; Volkow et al., 1997). Hence, clinical heterogeneity and the timing of testing relative to the last drug use may have a pronounced effect on reward-related tasks. Some recent hybrid models have begun to integrate concepts across different stages of addiction (Blum et al., 2012; Leyton, 2007). The incentive salience hypothesis acknowledges that dispositional weaknesses in executive function may explain why only a subset of individuals exposed to addictive drugs go on to develop an addiction (Robinson and Berridge, 2008). The two-factor dopamine model by Leyton (2007) proposes that motivational circuitry is hyperactive in response to addiction-related cues, but that this may lead to a devaluation of non-drug-related appetitive cues over time, such that neural processing of natural rewards may be intact in the premorbid state but reduced in addicted groups.

## 3 Using fMRI to investigate the neural basis for addiction

The blood oxygen level dependent (BOLD) signal measured during fMRI provides an indirect marker of neural activity deriving from changes in cerebral blood flow, which in turn reflect the increased energy demands that result from neural activity. Given the focus on the psychological theories of addiction on dopamine transmission, it is important to recognise that the fMRI signal is several steps removed from the dopaminergic neurons of the reward network, such that inferences about changes in dopaminergic activity should be made with extreme caution.

The dopamine pathways originate in the dopaminergic midbrain nuclei, although these nuclei are difficult to visualise with fMRI (Düzel et al., 2009; Limbrick-Oldfield et al., 2012), and most studies focus instead on regions that receive inputs from the dopaminergic midbrain: the dorsal and ventral striatum, and multiple sectors of the prefrontal cortex. These regions are larger, less prone to physiological noise, and BOLD signal is thought to correlate best with local field potentials that reflect dendritic inputs to the region and activity of local interneurons (Logothetis, 2003). While changes in the functional activity of this ‘reward circuitry’ has been interpreted as a modulation of the underlying dopaminergic inputs, a region like the striatum receives many inputs and contains many neuromodulators besides dopamine. When interpreting fMRI results in terms of hypo- or hyper-activity, one must also be aware that fMRI is unable to discriminate between excitatory and inhibitory neural activity, and thus a region could be ‘hyperactive’ as a result of a net increase in inhibitory activity.

Fortunately, we are not interpreting fMRI results in isolation. A seminal multi-modal imaging study correlated PET measures of dopamine release to a rewarded task against event-related fMRI responses during reward anticipation in the same participants (Schott et al., 2008). Dopamine release in the ventral striatum predicted the magnitude of BOLD signal changes in both the dopaminergic midbrain and the ventral striatum. Translational data from experimental animals also help substantiate the interpretations of imaging results; for example by highlighting functional subdivisions in the striatum and PFC that are at the boundary of the spatial resolution of fMRI. This work associates the dorsal striatum primarily with the acquisition of response–reward associations (Balleine and O'Doherty, 2010; see also O'Doherty et al., 2004) and habit formation (Haber and Knutson, 2010; Yin and Knowlton, 2006) whereas the ventral striatum is implicated in reward-related anticipation and prediction, and response vigour (Balleine and O'Doherty, 2010; O'Doherty et al., 2004; Roesch et al., 2009). Similar dissociations may be present in PFC, with the medial orbitofrontal region and rostral portion of the anterior cingulate implicated in stimulus value representations, contrasting with dorsal anterior cingulate holding action–value associations (Rushworth et al., 2011).

## 4 Neural processing of reward in drug addiction

Hommer et al. (2011) provide an authoritative and insightful overview of neuroimaging data that bear on the reward deficiency and impulsivity hypotheses, published up until 2010. Their conclusion is that while PET evidence of reduced dopamine D2 availability and blunted stimulant-induced dopamine release in drug addiction strongly favours the reward deficiency hypothesis (Fehr et al., 2008; Martinez et al., 2004; Volkow et al., 1997, 2001; Volkow et al., 2007), the more numerous fMRI literature of reward processing comprises reports of increases and decreases in reward processing in substance use disorders in roughly equal measure. Recent papers have continued this pattern of inconsistency. Common to many fields of research in fMRI, a range of different tasks are used to investigate the neural basis of addiction. However, this cannot be the sole explanation of the observed differences, as hypo-reactivity and hyper-reactivity have been observed on ostensibly similar tasks. Consider two recent studies with the monetary incentive delay task (MIDT), a simple and standardised task developed to investigate reward-related processes in the ventral striatum, with a particular focus on reward anticipation. A study of adolescent smokers found a *lower* ventral striatal response during reward anticipation, compared to non-smokers, and a *negative* correlation with smoking frequency, in accord with the reward deficiency hypothesis (Peters et al., 2011). No group differences were found during outcome processing. However, in the first study to use the MIDT in cocaine dependence, Jia et al. (2011) observed *enhanced* bilateral ventral- and dorsal–striatal reactivity to both reward anticipation and reward outcome, and this hyper-reactivity predicted poorer treatment outcomes (self-reported abstinence, urine toxicology) at two-month follow-up. Even across studies in drug users with the same preferred substance, the direction of effect is seen to fully reverse across different studies; for example, in alcohol dependence (Beck et al., 2009; Bjork et al., 2008; Wrase et al., 2007) or cannabis users (Nestor et al., 2010; van Hell et al., 2010) (see Hommer et al., 2011 for full descriptions of these studies).

Some of the inconsistencies in the field are likely due to clinical or demographic factors that act as moderators, such as the differences between classes of drug (e.g., stimulants vs. opiates) (McNamara et al., 2010), gender (Potenza et al., 2012), or treatment-seeking status (Stippekohl et al., 2012). Inclusion criteria are obviously important; for example, the target group in the Peters et al.'s (2011) study were adolescents who reported smoking at least one cigarette in the last 30 days, while Jia et al. (2011) included cocaine users seeking treatment for dependence. Thus, the similarities in task design must be weighed against major differences in the stage and severity of addiction. Even in studies of users preferring the same drug, there can be dramatic differences in inclusion criteria. For example, in studies of alcohol-dependence, Beck et al. (2009) and Wrase et al. (2007) excluded participants with a history of illicit drug use, while Bjork et al. (2008) included illicit drug users. Length of abstinence is similarly variable and known to impact on neural responses to drug-related cues (David et al., 2007; Fryer et al., 2012).

**Fig. 1:**
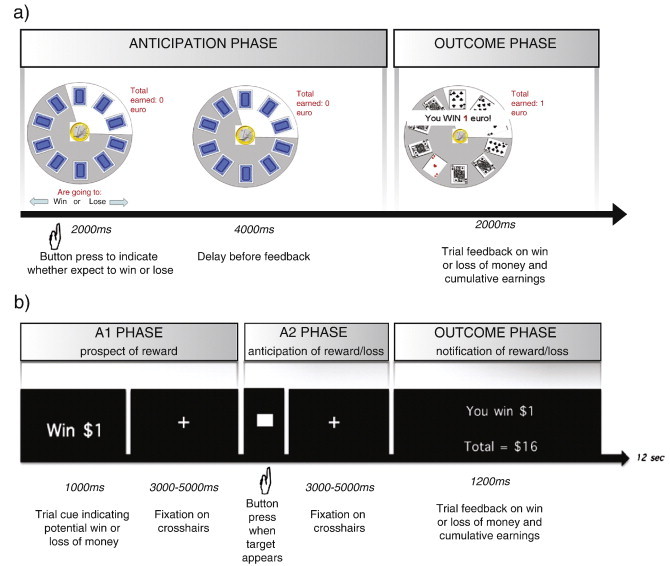
Structural differences between two typical tasks used to investigate appetitive processing in addiction. a) A guessing task adapted from Yacubian et al. (2006), and used by van Holst et al. (2012) in the study of pathological gamblers. On each trial, participants were presented with a representation of the chances (30% or 70%) and amounts (€1 or €5) that they could win or lose. Subjects indicated whether they expected to win or lose with a button press. This was followed by a 4 second anticipation period followed by the trial outcome. A win occurred when a red ace was within the highlighted area. b) A monetary incentive delay task adapted from Knutson et al. (2001) used by Balodis et al. (2012). On each trial, participants were presented with the amount they could win or lose (the first anticipation, A1, phase). During the second anticipation (A2) phase, the participants pressed a button when a target appeared. If the response was quick enough, they won, or avoided losing, the cued amount, with titration of the reaction time to ensure participants were correct on 66% of trials.

Several variables in fMRI task design may also influence the direction of effects. Given the temporal properties of the BOLD signal, the trial structure may be of even greater significance than clinical heterogeneity, and was favoured as one of the main explanations for the inconsistent results reviewed by Hommer et al. (2011). Even within a seemingly standardised task like the MIDT, one may be surprised by the number of subtle variants that exist (see Fig. 1). Some reports maximise power in the appetitive contrast by only comparing rewarded cues against non-rewarded cues (Peters et al., 2011), whereas others include a loss condition (Balodis et al., 2012a; Beck et al., 2009; Bjork et al., 2011; Jia et al., 2011; Nestor et al., 2010; Wrase et al., 2007). Studies in healthy volunteers have clearly established the sensitivity of the striatal response to these contextual factors (Bunzeck et al., 2010; Hardin et al., 2009; Nieuwenhuis et al., 2005): for example, a zero-win outcome is processed differently in tasks where loss can be sustained. The choice of baseline condition will be a crucial determinant of whether group differences reflect apparent increases or decreases in activity. Looking at the MIDT literature so far discussed, the baseline used is often a neutral cue or outcome (Bjork et al., 2008; Jia et al., 2011; Peters et al., 2011; Wrase et al., 2007), but some studies take alternative baselines like the inter-trial interval (Nestor et al., 2010).

A more subtle issue exists in the trial structure of the task, in order to de-couple various psychological phases within a trial. In a typical appetitive task, four stages might occur (see Fig. 1): the presentation of a motivational cue that creates a positive, neutral, or negative expectation on that trial, the participant's behavioural response to that cue, an anticipatory stage (either a delay or a more interesting spin of a wheel), and finally the delivery of the outcome. Without adequate temporal separation of these phases (‘jitter’), group differences that are *detected* at outcome could in fact be driven by abnormalities bleeding across from the earlier phases, given the sluggish timecourse of the BOLD signal. Thus, changes in deliberation or risk-taking during the response phase, or changes in anticipatory processing, could confound outcome effects. As is widely known from work in experimental animals, dopaminergic signalling is likely to shift over the course of an appetitive task from the reward itself (i.e., the outcome phase) to stimuli that predict those rewards (i.e., the cue or anticipation phases). In the plethora of variants used in addictions research, the overall task duration can be shortened considerably by removing these jittered intervals and presenting at least some of the stages in quick succession (Beck et al., 2009; Jia et al., 2011; Nestor et al., 2010; Wrase et al., 2007). Conversely, other experiments have specifically inserted jittered windows to isolate, for example, motor-preparatory activity (known to recruit striatal regions) from reward anticipation (Balodis et al., 2012a; Bjork et al., 2011; Peters et al., 2011), or reward anticipation from reward outcome. Nevertheless, even taking this critical issue into account, in the studies that have jittered anticipation and outcome we can still see variability of whether group differences occur in anticipation (Beck et al., 2009; Peters et al., 2011; Wrase et al., 2007) or at reward outcome (Balodis et al., 2012a; Jia et al., 2011).

Another methodological point concerns the nature of the reward itself. The majority of studies of reward processing in drug addiction have utilised monetary reinforcement (including all studies with the MIDT). While the reasons for using monetary reinforcement across experimental psychology is clear (e.g., clear motivational effects, and the ability to model gains and losses within the same domain), money is a complex reinforcer. First, its value is learned, albeit early in life such that by adulthood the brain may regard money on a par with primary rewards. Its subjective value differs between individuals as a function of wealth (the ‘Bernoulli Effect’; see Tobler et al., 2007 for neural instantiation of this phenomenon), and is derived from its capacity to be exchanged for other goods of value (i.e., it is fungible). This creates a specific issue in studies of addiction, as money acquired in an experimental setting can then be exchanged subsequently for the drug of abuse, placing it at a somewhat ambiguous level of incentive salience. It is unclear whether it should be regarded as an addiction related cue, or a natural reward.

Given these difficulties with the use of monetary reinforcement in studies of drug addiction, one useful design to gain leverage between the competing psychological hypotheses is to employ non-financial (and non-drug-related) appetitive cues such as erotica or pleasant tastes. These studies have generated a more uniform pattern of hypo-reactivity in reward-related regions (Asensio et al., 2010; Garavan et al., 2000; Wexler et al., 2001). For example, using erotic images from the International Affective Picture Series in a relatively large group of male cocaine dependent subjects, Asensio et al. (2010) found a broadly similar network recruited by appetitive cues in the two groups, but reduced activation in the dorsal and ventral striatum and dorsomedial PFC in the cocaine group. These studies support the reward deficiency hypothesis, but can also be accommodated in variants of incentive salience (e.g., Leyton, 2007) that allow the sensitisation of drug-related cues to drive an attenuation in the response to natural reinforcers.

## 5 Pathological gambling

Since its inclusion in the DSM-III in 1980, pathological gambling has been grouped in the impulse control disorders, alongside kleptomania, pyromania and trichotillomania. The DSM5 proposal to re-classify it in the addictions category (Holden, 2010; Petry, 2010) has been prompted by several lines of research, including empirical evidence for a shared addiction vulnerability (e.g., Lind et al., 2012; Lobo and Kennedy, 2009; Slutske et al., 2000) and substantial similarities in the neural underpinning revealed primarily by fMRI (Leeman and Potenza, 2012; Potenza, 2008). As well as being the ‘flag-bearer’ for the *behavioural* addictions, we believe that pathological gambling also provides an important model for the addictions field more broadly, for at least two reasons. The first reason concerns the intractable ‘chicken and egg’ problem in addictions research (see Ersche et al., 2010; Verdejo-García et al., 2008). The chronic consumption of most drugs of abuse is associated with gross structural changes in the brain, such that the neural signatures of premorbid vulnerability cannot be dissociated from changes that have taken place as a consequence of the drug use. Such overt neurotoxicity should be absent in pathological gambling, and indeed two recent studies using voxel-based morphometry were unable to detect significant changes in grey or white matter volumes in pathological gamblers (Joutsa et al., 2011; van Holst et al., 2012a), contrasting with the dramatic and widespread reductions in grey matter in a matched group with alcohol dependence (Chanraud et al., 2007; van Holst et al., 2012a). A further complication arising from the same effect is that group comparisons of functional activity against healthy controls can be confounded by structural volume differences, where such effects are present in drug addictions. Admittedly, in pathological gamblers, the regular cycle of winning and losing may conceivably engender more subtle neuro-*adaptive* changes that might not be readily detectable with structural imaging protocols. Nevertheless, phenotypic similarities between pathological gamblers and groups with drug addictions, such as on trait impulsivity and neuropsychological probes of risky decision-making, may be more aligned with vulnerability mechanisms than the neurotoxic sequelae of chronic drug use.

The second kind of insight that may be afforded from research on pathological gambling concerns that nature of reinforcement in neuroimaging studies. The experience of financial wins, and instrumental behaviour to obtain those outcomes, are the defining features of gambling, and key conditioning stages in the development of pathological gambling (Blaszczynski and Nower, 2002). Thus, in research in individuals with pathological gambling, the abused ‘commodity’ is now congruent with the experimental tractability of monetary reinforcement in reward-based tasks. Unfortunately, the growing literature that has used monetary tasks in pathological gamblers suffers from the same heterogeneity that we have described above in drug addiction. A groundbreaking early study from Reuter et al. (2005) used a two-choice card guessing task to compare the brain response to wins versus losses in pathological gamblers. Signal change in the ventral striatum and ventral medial PFC (vmPFC) was reduced in the pathological gamblers, and correlated negatively with gambling severity. However, this study did not employ a neutral outcome condition, and only modelled outcome-related activity on each trial. The baseline used was loss outcomes, hence any group differences could be driven by either changes in loss- or gain-related processing. A somewhat similar pattern was reported in the ventrolateral PFC for feedback on a reversal learning task in pathological gamblers (de Ruiter et al., 2009).

In more recent studies that tease apart the temporal dynamics within a trial, a more complicated pattern emerges. Van Holst et al. (2012b) used a probabilistic choice game that varied both the magnitude and probability of the potential reward across trials, and modelled brain responses during the anticipation phase (see Fig. 1). Pathological gamblers displayed a greater response to the magnitude contrast (win 5 euros versus win 1 euro) in the dorsal striatum, compared to controls, and the dorsal striatum and OFC also tracked gain-related expected value to a greater extent in the pathological gamblers. However, in a contemporaneous paper, Balodis et al. (2012a) reported a reduction in fronto-striatal circuitry using the MIDT in pathological gamblers. Their task enabled temporal separation of anticipation and outcome, and during anticipation, the gamblers displayed reduced activity in ventral striatum and vmPFC across all expectancy conditions (gains and losses). On receipt of a financial gain, the pathological gamblers also showed decreased vmPFC activity.

The disparities between these two results are initially baffling, but there are some important design differences between the experiments that may provide clues of wider relevance to the addictions field. First, while the tasks both employed monetary reinforcement, the precise form of presentation was very different (Leyton and Vezina, 2012): Van Holst et al. (2012b) used realistic playing cards and images of actual money (see Fig. 1), whereas Balodis et al. (2012a) did not involve a realistic gambling scenario, and stated the amount to be won or lost in simple text format. A pathological gambler may conceivably experience the first task as evocative of real play, whereas the second task may not be closely associated with the addictive behaviour despite the availability of monetary reinforcement. Leyton and Vezina (2012) suggest that the processes of incentive salience may be specific to just a narrow set of stimuli that are intimately related to the addiction. There are also further differences between the two tasks besides the cues, including trial timings and analysis. Van Holst et al. (2012b) used a contrast of large reward anticipation against small reward anticipation, while Balodis et al. (2012a) used a categorical contrast with a neutral anticipation period as a baseline. Clearly group differences in the processing of magnitude changes during anticipation are distinct from group differences in processing of the anticipation of a rewarding vs. neutral outcome.

Moreover, the group differences described by van Holst et al. (2012b) and Balodis et al. (2012a) studies refer to distinct sectors of the striatum. The enhanced *dorsal* striatal activity in the van Holst study (2012b) could be interpreted as evidence that gamblers are inclined to form action–outcome associations during gambling, whereas the hypo-responsiveness in the *ventral* striatum in the Balodis et al.'s (2012a) study could indicate inflexibility to update reward values (for discussion see Balodis et al., 2012b; van Holst et al., 2012c). Hence, the role of separate striatal subdivisions may be critical in interpreting these results.

Other neuroimaging studies suggest that group differences between pathological gamblers and controls may depend on specific task conditions. An fMRI study of blackjack indicated enhanced inferior frontal gyrus and thalamus activity in problem gamblers only during high-risk trials; no group differences were observed during low-risk trials (Miedl et al., 2010). These results were corroborated with EEG, where problem gamblers showed a positive amplitude over frontal cortex on high-risk rewarded trials, whereas no group differences were seen on low-risk trials (Hewig et al., 2010; Oberg et al., 2011). These results are in line with the suggestion by Leyton and Vezina (2012), that the processes of incentive salience in gamblers may become very specific to a narrow set of high-risk opportunities.

The specific relevance of monetary reinforcement to pathological gambling also allows a direct comparison of the ‘addictive’ reward against natural rewards, such as food or sexual stimuli. This formed the rationale behind a third recent experiment in pathological gambling, comparing the neural response to financial rewards and erotic visual rewards, using an incentive delay task (Sescousse et al., 2010). During anticipation, pathological gamblers showed a *reduced* neural response in the ventral striatum for erotic rewards compared to controls, consistent with the study in cocaine dependence described above (Asensio et al., 2010). During anticipation, there was no difference in the response to financial rewards. However, during the outcome phase, the neural response to financial outcomes was *increased* in the pathological gamblers compared to controls in the orbitofrontal cortex. This pattern of results is not well accommodated by any of the hypotheses for addiction outlined above, if taken on their own. Rather, the data support a two process model, either where the hyper-reactivity to addictive rewards drives an attenuation of the response to natural rewards (Leyton, 2007) or where initial reward deficiency is supplemented by an incentive salience process to addiction-related cues (Blum et al., 2012). Note that either mechanism assumes an incentive sensitisation process driven only by behaviour, with no exogenous dopamine input. A logical next step to separate these possibilities would be to identify a high-risk group for pathological gambling, such as first-degree relatives, to fully isolate the vulnerability markers.

The final recent study in pathological gamblers has adopted a computational approach to consider the neural representations of reward as a function of changes in the delay to the reward (temporal discounting) and the uncertainty of reward (probability discounting) (Miedl et al., 2012). The underlying behavioural phenomena are well-established: in problem gambling and drug addiction, there is increased discounting of delayed rewards (i.e., a preference for immediate reward) and a reduced discounting of uncertain rewards (i.e., less risk aversion) (Madden et al., 2009). The Miedl et al.'s (2012) experiment titrated the subjective value for both delayed and probabilistic choices for each individual, and these values were then reliably correlated with brain activity in the ventral striatum. The pathological gamblers showed greater value representations in ventral striatal in the temporal discounting task, but reduced value representations during the probability discounting task, compared to controls. These results implicate a distortion of the value functions relating rewards to time and uncertainty in problem gamblers, and these choice-based tasks converge on the same core pathophysiology as revealed by the motivational tasks in the work above.

## 6 Conclusion

From the complex picture that has been described above, it is important to recognise the robust localisation of the group differences in addiction to the reward-related circuitry comprising primarily the ventral striatum and medial PFC. It is the inconsistent direction of effects within this circuitry that forms the topic for discussion, representing as it does a major obstacle in the use of fMRI data for adjudicating between the psychological theories of addiction. One view might be that the available data clearly highlight an *impairment* in this system, and that the exact direction may be relatively unimportant. However, our opinion from surveying this corpus of research is that relatively subtle methodological decisions at the level of the task design, trial structure, and analysis can have a critical impact on the group differences observed. While these principles are well recognised in imaging textbooks, we would encourage researchers to be cognisant of the idea that such decisions may drive group differences in entirely opposing directions, and to consider these methodological influences before advocating support for an underlying theory. Several factors are likely to be important in this regard: 1) inclusion of positive, negative and neutral outcomes in the same task, or comparison of only positive and neutral conditions. Neutral cues or outcomes (which constitute the most standard baseline condition) are known to be processed differently in these two contexts (e.g., Nieuwenhuis et al., 2005); 2) the trial timings with regard to temporal segregation of choice/response, anticipation, and outcome-related processing. While it is tempting to prioritise shorter task lengths, and early work in this area did often collapse across some phases, this is likely to ultimately hinder consistency; and 3) the nature of the appetitive cues; and even within tasks using the same apparent cue type (e.g., monetary outcomes), there may be a meaningful influence of the graphical representation, such as coin images versus text feedback of monetary outcomes (see Fig. 1), which may be sufficient to drive addiction-related processing.

Given these design issues, on-going functional neuroimaging research on drug addiction would benefit from a broader range of study designs. To best differentiate between the dominant psychological models, three kinds of design are particularly powerful. It is highly likely that drug-related cues are processed differently from other non-addiction-relevant appetitive cues in addicted individuals, although very few studies have directly compared these classes of cue in the same design (see Sescousse et al., 2010 for an exception). Given the complexities with the use of money as a fungible reinforcer in drug addiction, a fruitful approach is to measure the neural responses to primary rewards such as erotica or pleasant tastes (Asensio et al., 2010; Garavan et al., 2000; Horder et al., 2010). Second, it is difficult to disentangle the dominant psychological theories in studies in drug addicted groups, where the premorbid vulnerability factors (such as reward hyposensitivity) may have already been altered by the transitional processes into addiction, including the neurotoxic and neuroadaptive changes induced by chronic drug use. Research in high-risk groups by virtue of family history, genotype, or personality dispositions such as trait impulsivity, is required to isolate markers of vulnerability per se, and research on pathological gambling may also be useful in this regard. Third, with the historical emphasis on dopamine focussing work on the appetitive system, far less neuroimaging work has sought to quantify aversive processing in addiction. Nevertheless, a number of psychophysiological studies have described an attenuated response to aversive cues in addictions, including deficits in Pavlovian fear conditioning (Brunborg et al., 2010; McGlinchey-Berroth et al., 1995, 2002), and the error-related negativity (Franken et al., 2007). While preliminary fMRI work has corroborated a blunting of loss-related activity in the striatum, anterior cingulate and insula in drug addiction (de Ruiter et al., 2012; Forman et al., 2004; Kaufman et al., 2003), these studies are yet to consider issues such as reinforcer type and stage of processing (e.g., anticipation versus outcome) that are raised in the far more numerous studies of appetitive processing.

Finally, we would emphasise the insights that are offered by research on individuals with pathological gambling within the addictions framework. Studies of pathological gamblers can reveal the neural underpinnings of addiction in an illness that is not confounded by the pronounced neurotoxic effects that result from substance abuse; indeed recent VBM experiments in pathological gamblers have detected no significant structural differences (Joutsa et al., 2011; van Holst et al., 2012a). In addition, we have highlighted some of the complexities with using money as the reinforcer in studies of drug addiction; namely that it is a complex learned reinforcer that is exchangeable (at least in principle) for the drug of abuse. Given the practical utility of using monetary reinforcement in neuroimaging tasks, pathological gambling represents a condition where there is direct convergence of the task reinforcer and addictive cue: for pathological gamblers money *is* an addiction-related cue. The fMRI literature on pathological gambling has matured in the past two years, and while future work is likely to elaborate on important clinical predictors like length of abstinence and treatment-seeking status, which have received little consideration so far, significant progress has already been made. Importantly, abstinence is not required for the investigation of pathological gambling, due to the lack of intoxication effects. Therefore, this could afford investigators the opportunity to investigate all stages of the addiction cycle. As pathological gambling is re-classified with the substance addictions in the forthcoming DSM5, we anticipate further lines of convergence from pathological gambling to drug addiction and vice versa.
